# P-1021. Risk Factors Associated with Non-antibiotic Medication Use among Persons with Clostridiodes difficile, Davidson County, Tennessee, 2018–2022

**DOI:** 10.1093/ofid/ofaf695.1217

**Published:** 2026-01-11

**Authors:** Malakai Miller, Sarah Petersen, Samantha Mathieson, Christopher Wilson, Melphine Harriott

**Affiliations:** Tennessee Department of Health, Nashville, Tennessee; Tennessee Department of Health, Nashville, Tennessee; Tennessee Department of Health, Nashville, Tennessee; Tennessee Department of Health, Nashville, Tennessee; TN Department of Health, Nashville, Tennessee

## Abstract

**Background:**

*Clostridioides difficile* infection (CDI) is one of the most common healthcare-associated infections. Risk factors include older age, sex, antibiotic exposure, and healthcare facility exposure. As part of the Emerging Infections Program’s CDI surveillance, we examined the 5 most common comorbidities and analyzed possible associations between select patient characteristics and non-antibiotic medication use among Davidson County, TN CDI cases.

**Methods:**

Surveillance data from 2018–2022 were examined for fully abstracted incident CDI cases in Davidson County. An incident case was defined as *C. difficile*-positive stool test from a person ≥1 year old with no prior positive test within 8 weeks. Simple statistics and odds ratios were calculated using SAS v9.4.

**Results:**

Of the 1,893 cases, 4.0% were healthcare-facility onset, 24.3% community-onset healthcare-facility associated, and 71.7% community acquired. 62.3% of cases were female. 53.7% of cases were adults (18–64 years), 40.0% were elderly (≥65 years), and 6.2% were children (1–17 years). The 5 most common comorbidities were diabetes (DM), chronic kidney disease (CKD), chronic obstructive pulmonary disease (COPD), diverticular disease (DD), and obesity. We found correlations between males and DM (OR 1.5; 95% CI 1.2–1.9) and CKD (OR 1.7; 95% CI 1.3–2.2). Children were less likely to use PPIs (OR 0.3; 95% CI 0.2–0.6) while the elderly were more likely to use PPIs (OR 1.6; 95% CI 1.2–1.9) than adults. Children were less likely to use non-inhaled steroids than adults (OR 0.6; 95% CI 0.3–0.9).

**Conclusion:**

Males were significantly associated with DM and CKD. Children and the elderly were significantly associated with PPI use, and children with non-inhaled steroid use. Understanding associations between non-antibiotic medication use and CDI can inform better clinical practices and guide healthcare interventions, especially for vulnerable populations such as the elderly and those with specific comorbidities. Continued surveillance and research are essential to address the complexities of CDI and enhance patient outcomes. Further analyses in combination with other data sources, including matched cohorts of non-CDI populations, will be essential to determine the generalizability of these preliminary findings.

**Disclosures:**

All Authors: No reported disclosures

